# Intestinal Claudin-7 deficiency impacts the intestinal microbiota in mice with colitis

**DOI:** 10.1186/s12876-022-02100-8

**Published:** 2022-01-17

**Authors:** Yuhan Ding, Kun Wang, Chang Xu, Mengdi Hao, Huimin Li, Lei Ding

**Affiliations:** 1 Department of Oncology, Beijing Shijitan Hospital, Capital Medical University, Tieyilu 10, Yangfangdian, Haidian District, Beijing, 100038 China; 2 Department of Hepato-Pancreato-Biliary Surgery, Key Laboratory of Carcinogenesis and Translational Research, Ministry of Education/Beijing, Peking University Cancer Hospital and Institute, Beijing, 100142 China

**Keywords:** Claudin-7, Colitis, Intestinal microbiota, Tight junction, Microbiota dysbiosis

## Abstract

**Background:**

Intestinal epithelial cells form a physical barrier that protects the intestine against the intestinal microbiota through tight junctions (TJs) and adhesive junctions, while barrier disruption may lead to inflammatory bowel disease (IBD). Claudin-7 (Cldn7) has been implicated in this protection as an important member of TJs. Here, we experimentally study the effect of Cldn7 deletion on intestinal microbiota in colitis.

**Methods:**

Colitis model was established based on inducible intestinal conditional Cldn7 gene knockout mice (Cldn7fl/fl; villin-CreERT2), by feeding with dextran sodium sulfate (DSS). AB-PAS staining and immunohistochemical staining of Muc2 mucin were used to detect the effect of Cldn7 deficiency on the mucus layer of mice with colitis, and fluorescence in situ hybridization was used to detect how Cldn7 promotes spatial separation of the gut microbiota from the host. The microbiota population was characterized by high-throughput 16S rRNA gene sequencing of DNA extracted from fecal samples.

**Results:**

Compared with the controls, Cldn7 knockout increased susceptibility to colitis, including greater degree of weight loss, colon shortening, and a significantly higher disease activity index score. DSS-treated Cldn7 knockout mice promoted the migration of bacteria to the intestinal epithelium to some extent by damaging the intestinal mucus layer. Sequencing of 16S rRNA showed that DSS-treated Cldn7 knockout mice reduced the gut microbiota diversity and had greater relative abundance of *Escherichia coli*. LEfSe analysis indicated that *Escherichia coli* may be the key bacteria in Cldn7 knockout mice during DSS-induced colitis. Furthermore, the Tax4Fun analysis predicted that DSS-treated Cldn7 knockout mice enriched for microbiota impacting infectious diseases, immune system and metabolic functions.

**Conclusions:**

Our data suggests an association between intestinal Cldn7 knockout and microbiota dysbiosis during inflammatory events.

## Background

Inflammatory bowel disease (IBD), comprising ulcerative colitis and Crohn's disease, is characterized by chronic inflammation of the gastrointestinal tract and mucosal tissue damage [1]. The etiology of IBD is not yet clear, but studies so far have shown that intestinal barrier dysfunction, abnormal immune response, and microbiota dysbiosis are closely related to intestinal inflammatory imbalance [2].

Intestinal epithelial cells (IECs) form a physical barrier against the gut microbiota through tight junctions (TJs) and adhesion junctions [3]. The changes in TJ integrity and increase in intestinal mucosal permeability precede and exacerbate intestinal inflammation development, allowing the microbiota to invade and stimulate immune cells to produce inflammatory antigens [4, 5]. Claudins are important TJ components, and their abnormal expression can lead to reduced cell adhesion, structural damage, and impaired epithelial and endothelial cell function, as well as act as signaling proteins involved in inflammation, cell proliferation and differentiation [6, 7]. Claudin-7 (Cldn7) is a member of the claudin protein family, which is unique in that it has a stronger basolateral membrane distribution than other claudins, which localize primarily to apical TJs in the intestinal epithelium [8]. Previous studies have shown that Cldn7 knockout leads to an imbalance in the paracellular flux of intestinal small organic solutes, promotes the degradation of intestinal extracellular matrix, enhances of mucosal immunity, and induces intestinal inflammation [9]. Three different Cldn7 knockout mice models have been developed, among which the Cldn7 inducible conditional knockout mice we established can spontaneously develop atypical hyperplasia and intestinal adenoma [9–11]. Consequently, these results suggest that Cldn7 plays a critical role in shaping the intestinal mucosal barrier and maintaining intestinal homeostasis.

It is well known that the intestinal mucus barrier spatially separates the gut microbiota and the host, which prevents unnecessary conflict to maintain the symbiotic relationship [12]. Microbiota dysbiosis is characterized by microbial population, diversity, spatial, number or function change, which has been identified as important factors leading to inflammation and damage to the colonic epithelium in IBD patients, which in turn will lead to subsequent disease progression [13, 14]. Recent evidence indicated that the epithelial hyperpermeability as an initiating factor for microbiota dysbiosis that eventually led to pathological consequences of IBD [15]. Although Cldn7 is considered to be related to maintaining the intestinal epithelial permeability and inhibiting colonic inflammation, we lack an understanding of how Cldn7 modulates the intestinal microbiotaand thus affects the development of colitis.

In this study, we used dextran sulfate sodium (DSS) administration to establish experimental colitis in inducible conditional Cldn7 knockout mice. Our aim was to experimentally examine the effect of Cldn7 deletion on intestinal microbiota in colitis. For the first time, our data suggests an association between intestinal Cldn7 knockout and microbiota dysbiosis during inflammatory events.

## Methods

### Animals

Inducible intestinal conditional Cldn7 gene knockout mice (Cldn7fl/fl; villin-CreERT2) were generated using the Cre/Loxp system as reported previously [16]. All mice were maintained under specific pathogen-free conditions in the animal facilities of Beijing Shijitan Hospital. The mice were given a standard chow diet and water ad libitum and were used for subsequent experiments at 8–10 weeks old. The animal experiments complied with the ARRIVE guidelines and were approved by the Animal Ethics Committee of the Beijing Shijitan Hospital Institutional Review Board.

### Establishment of DSS-induced colitis

The DSS-induced colitis mouse model was established as previously described [17]. CreERT2 and littermate control CreW mice were fed 2.5% DSS (molecular weight, 36–50 kDa, MP Biomedicals, LLC; Solon, OH, USA) for 7 days. Unchallenged animals received tap water. The disease activity index (DAI) scoring criteria is based on three parameters such as body weight, diarrhea and bloody stool. All mice were euthanized, removed the colon, measured the length of the colon, weighed the spleen, separated the colonic tissues into several segments for paraffin embedding or cryopreservation.

### HE and AB-PAS staining

The intestinal tissues of all mice were washed in PBS and fixed in Carnoy's fixative (Solarbio, Beijing, China) at 4 °C. After dehydration and clearing, the tissues were immersed in wax and then cut into 5-µm-thick sections. The sections were then dewaxed and stained with HE or AB-PAS Stain Kit (Solarbio, Beijing, China). Histopathology scores were evaluated by pathologists blinded to the experimental grouping according to the criteria of previous articles that considers four parameters (inflammation, extent, crypt damage and regeneration) [18].

### Immunohistochemistry

Immunohistochemical staining was used to detect the expression of Muc2 mucin (1:2000, Abcam, Cambridge, MA, USA). The tissue slides underwent dewaxing, rehydration, antigen retrieval, and incubated with 3% hydrogen peroxide (H_2_O_2_) to inactivate endogenous peroxidase activity. The sections were blocked with 3% bovine serum albumin (BSA) for 1 h at RT, and incubated with the corresponding primary antibodies and secondary antibody. DAB was used as the final chromogen and hematoxylin counterstained the nuclei for immunohistochemistry.

### Fluorescence in situ hybridization (FISH)

Large intestinal tissues were prepared for FISH analysis by fixation in Carnoy's fixative, followed by embedding in paraffin as described above. Tissues were sectioned at a thickness of 5 µm and hybridized with a universal bacterial probe directed against the 16S rRNA gene: (EUB338 probe: *5′-GCTGCCTCCCGTAGGAGT-3′*; control probe: *5′-ACTCCTACGGGAGGCAGC-3′*). Fluorescence analysis was performed with a Nikon A1 laser confocal microscope (Nikon, Tokyo, Japan).

### DNA extraction

Total genomic DNA from samples was extracted using the CTAB method. DNA concentration and purity were monitored on 1% agarose gels. According to the concentration, DNA was diluted to 1 ng/µL using sterile water [19].

### Amplicon generation

16S rRNA genes of distinct regions were amplified using specific primers (16S V4: 515F-806R) with barcodes [19]. All PCRs were carried out with 15 µL of Phusion^®^ High-Fidelity PCR Master Mix (New England Biolabs), 2 µM forward and reverse primers, and approximately 10 ng of template DNA. Thermal cycling consisted of initial denaturation at 98 °C for 1 min, followed by 30 cycles of denaturation at 98 °C for 10 s, annealing at 50 °C for 30 s, and elongation at 72 °C for 30 s, and a final extension at 72 °C for 5 min [19].

### Library preparation and sequencing

An equal volume of 1X loading buffer (containing SYB green) was mixed with the PCR products, and electrophoresis was performed on a 2% agarose gel for detection. PCR products were mixed in equidensity ratios. Then, mixed PCR products were purified with a Qiagen Gel Extraction Kit (Qiagen, Germany) [19]. Finally, purified amplicons were pooled in equimolar and paired-end sequenced on an Illumina NovaSeq platform according to the standard protocols [20].

### Bioinformatic analysis

Paired-end reads with overlap were merged to tags. And tags were clustered to Operational taxonomic unit (OTU) at 97% sequence similarity. Representative sequences for each OTU were screened for further annotation. For each representative sequence, the Silva database (http://www.arb-silva.de/) [21] was used based on the Mothur algorithm to annotate taxonomic information. To study the phylogenetic relationship of different OTUs and the difference in the dominant species in different samples (groups), multiple sequence alignment was conducted using the MUSCLE software (V3.8.31, http://www.drive5.com/muscle/) [22]. OTU abundance information was normalized using a standard sequence number corresponding to the sample with the fewest sequences. Subsequent analyses of alpha diversity and beta diversity were all performed based on these output-normalized data.

Alpha diversity was applied to analyze the complexity of species diversity for a sample, including Observed-species, Chao1, Shannon, Simpson and ACE indices. Beta diversity analysis was used to evaluate differences in species complexity among samples. Alpha diversity and beta diversity were calculated with QIIME (V1.7.0) and displayed with R software (V2.15.3). Anosim (Analysis of similarities) and Adonis analysis use the Anosim function and Adonis function of the vegan software package of R software, respectively. To identify biomarkers with differentiating abundance in the different treatments, the LDA Effect Size (LEfSe: Linear discriminant analysis Effect Size) algorithm was used. Tax4Fun analysis was conducted to predict microbial functional profiling. Tax4Fun is an opensource R package that predicts the functional capabilities of prokaryotic communities based on 16SrRNA data sets. And heat map of the Kyoto Encyclopedia of Genes and Genomes (KEGG) level1/2 functional pathways [23–25] was carried out by R package.

### Statistical analysis

Data were expressed as the mean ± SEM and the differences among experimental groups were analyzed using GraphPad Prism 8.0 software (La Jolla, CA, USA). The significant difference between two groups was evaluated by the two-tailed unpaired Student’s t-test or Mann–Whitney U test analysis. Differences among three or more comparisons were analyzed using one-way ANOVA. Bioinformatic analyses were performed in R software. *P* < 0.05 was considered to indicate a certain statistically significant difference, and represented as *P < 0.05, **P < 0.01, ***P < 0.001, and ****P < 0.0001.

## Results

### Evaluation of the disease model

**Fig. 1:**
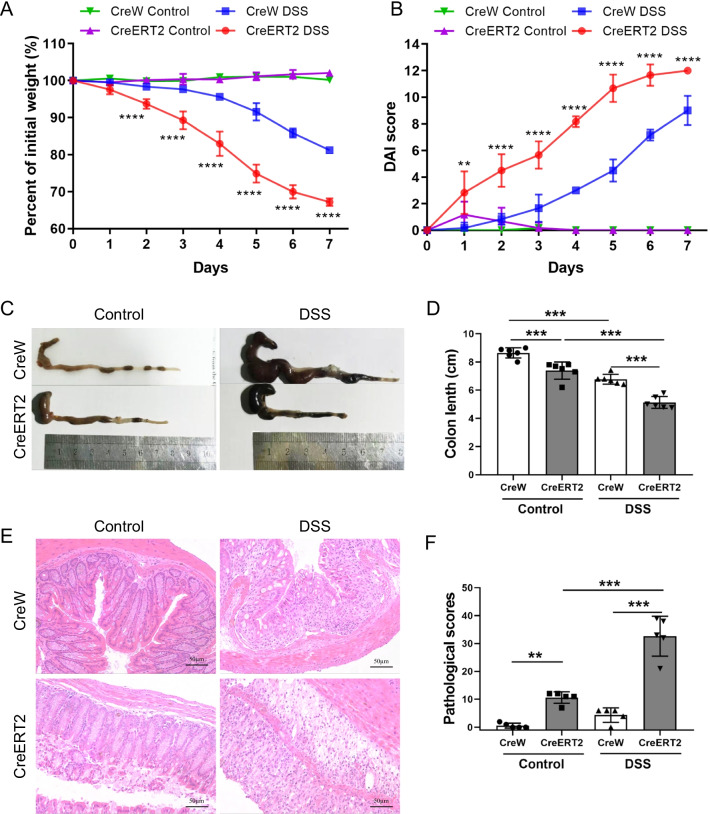
Intestinal Cldn7 knockout mice showed increased susceptibility to DSS-induced colitis. **A** Changes in the body weights of each mouse were measured as percent of the initial weight at the start of the experiments (CreERT2 + DSS vs. CreW + DSS, ****P < 0.0001, n = 6). **B** The DAI score was recorded daily (CreERT2 + DSS vs. CreW + DSS, **P < 0.01, ****P < 0.0001, n = 6). **C**, **D** Gross morphology and measurements of colon length (***P < 0.001, n = 6). **E**, **F** Representative H&E-stained colon sections in each group and colonic pathological scores for inflammation, extent of injury, crypt damage and regeneration (**P < 0.01, ***P < 0.001, n = 5)

We used previously constructed inducible intestinal conditional Cldn7 gene knockout (Cldn7fl/fl; villin-CreERT2, abbreviated as CreERT2) and control (Cldn7fl/fl; villin-CreW, abbreviated as CreW) murine models, and intraperitoneally injected tamoxifen (1 mg in 100ul sterile sunflower oil, every 4 days) for 5 consecutive inductions, followed by drinking water for 7 days and 2.5% DSS treatment for 7 days to establish the experimental colitis model. Compared with CreW littermates exposed to DSS, CreERT2 mice developed more serious intestinal disease, weight loss, colon shortening, and a significantly higher DAI score (Fig. 1A–D). The histopathological slides also showed increased intestinal inflammation levels in DSS-treated CreERT2 mice, accompanied by necrosis of the intestinal epithelium, glandular crypt structure destruction and inflammatory infiltration (Fig. 1E, F). These results indicate that the absence of Cldn7 aggravates the intestinal damage induced by DSS.

### The effect of Cldn7 deletion on the translocation of intestinal bacteria

**Fig. 2:**
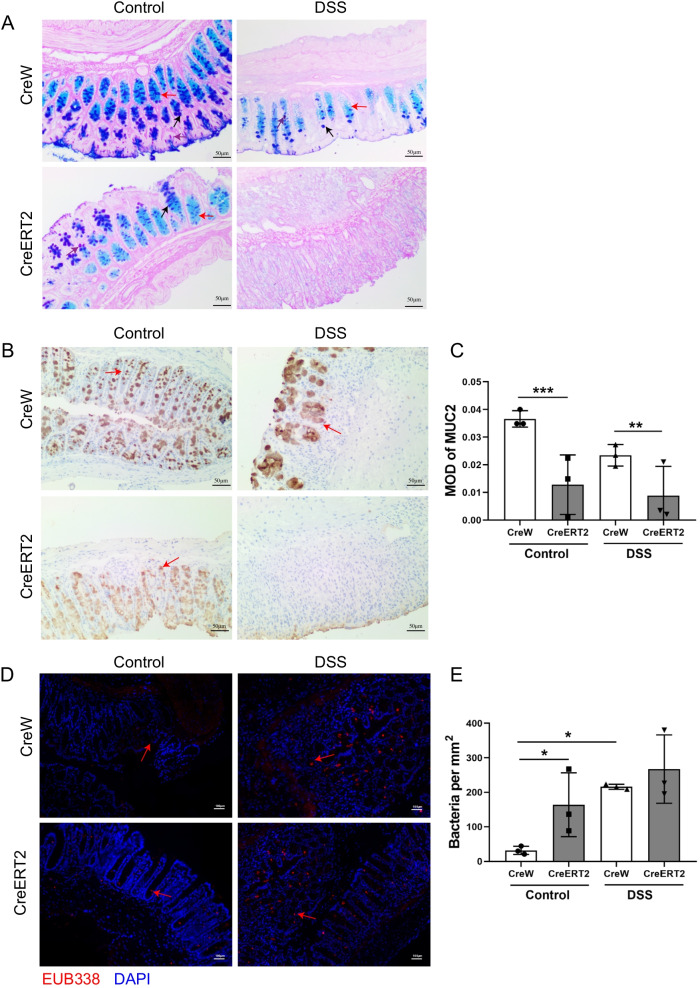
Effect of Cldn7 gene knockout on mucus layer and translocation of intestinal bacteria. **A** AB-PAS staining shows different kind of goblet cells containing: mixed (black arrow), neutral (purple arrow) and acid (red arrow) mucins. **B**, **C** Representative immunohistochemical images of Muc2 in control tissues and DSS-treated colonic tissues from CreW and CreERT2 mice, the red arrows represent positive cells. Right: the mean optical density (MOD) of Muc2 positive cells (**P < 0.01, ***P < 0.001, n = 3). **D**, **E** Bacteria were stained with EUB338 by fluorescent in situ hybridisation (FISH) in each group, the red arrows represent bacteria. Right: quantification the number of bacteria per mm^2^ in each group (*P < 0.05, n = 3)

Given that the mucus barrier is an important factor in protecting the intestinal epithelium from bacterial invasion, we used AB-PAS staining and immunohistochemistry to detect the effect of Cldn7 deficiency on the intestinal mucus layer of mice with colitis. The results of AP-PAS staining showed that the Cldn7 deficiency caused the intestinal mucosal structure disorder in mice with colitis, and observably reduced the number of goblet cells and mucins (Fig. 2A). These observations were consistent with the significantly reduced Muc2 mucin expression levels detected by immunohistochemistry in DSS-treated CreERT2 mice (Fig. 2B, C). Next, we used FISH to compare mucosal-related bacteria in CreW and CreERT2 mice (Fig. 2D, E). We detected increased numbers of bacteria in the colon wall of untreated CreERT2 mice compared with CreW mice, suggesting that bacterial translocation was facilitated in the absence of Cldn7. DSS-induced colitis further promoted bacterial translocation, although the total number of bacteria in direct contact with the intestinal epithelial surface showed no significant change between DSS-treated CreW and CreERT2 mice. These data indicate that the lack of Cldn7 disrupts the mucus barrier and increases penetrability to bacteria, but does not regulate overall numbers of colonizing bacteria during DSS-induced colitis.

### Loss of Cldn7 changes the diversity of intestinal microbiota

Fecal 16S rRNA sequencing was performed to compare microbial community diversity in four groups, including alpha diversity and beta diversity.

**Fig. 3:**
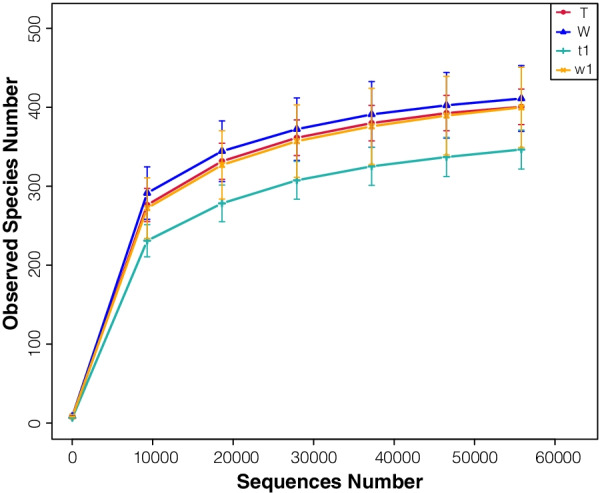
Rarefaction curve for community richness showed that sufficient sequencing depth was reached. Note: n = 6; T was untreated CreERT2 group, W was untreated CreW group, t1 was DSS-treated CreERT2 group, w1 was DSS-treated CreW group

**Fig. 4:**
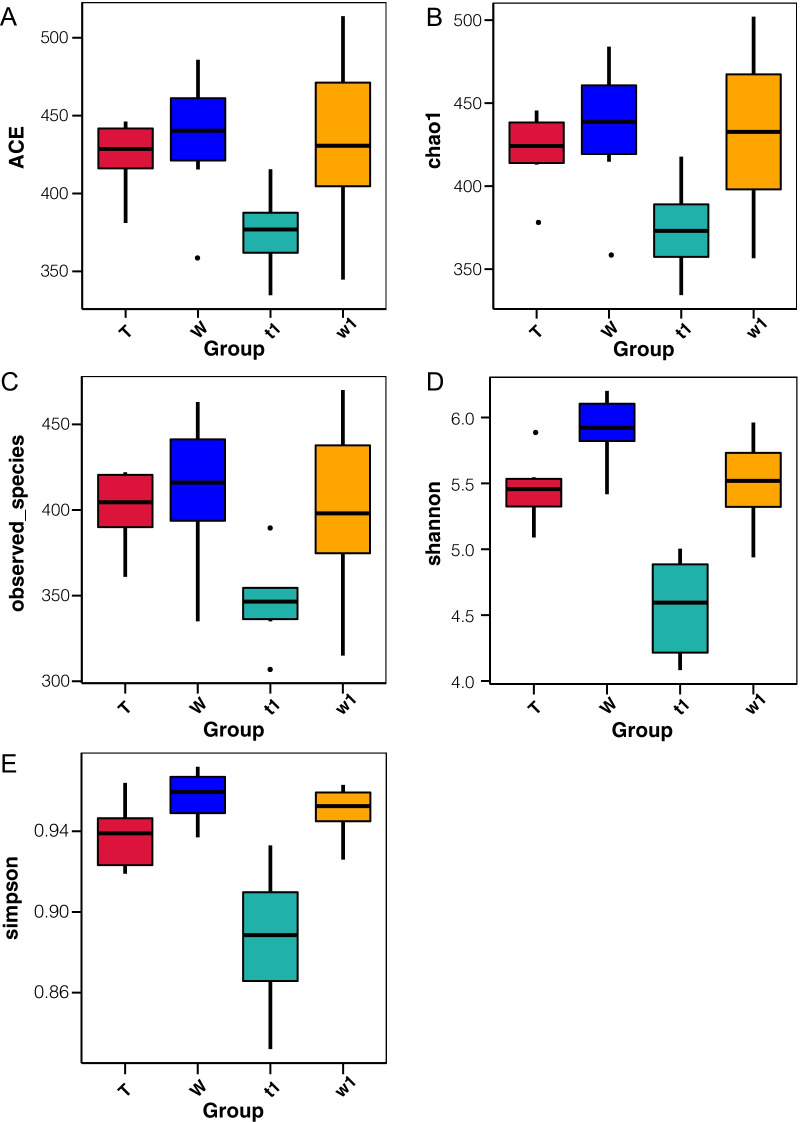
Alpha diversity of the gut microbiota in each group. Alpha diversity of the gut microbiota was significantly reduced in CreERT2 mice compared with that in CreW mice, according to the Shannon and Simpson indices. DSS treatment perturbed and reduced intestinal microbiota diversity. DSS-treated CreERT2 mice had reduced ACE, Chao1, Observed-species, Shannon and Simpson indices compared with DSS-treated CreW mice (**A**–**E**). Note: n = 6; T was untreated CreERT2 group, W was untreated CreW group, t1 was DSS-treated CreERT2 group, w1 was DSS-treated CreW group

**Fig. 5:**
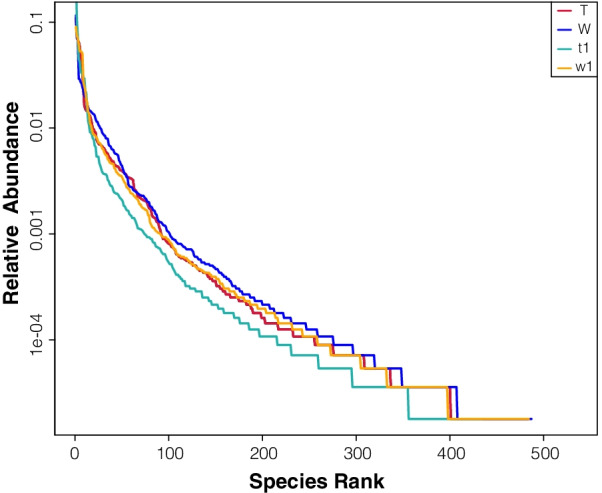
Rank-Abundance curve is used to reflect the species diversity of each group. Note: n = 6; T was untreated CreERT2 group, W was untreated CreW group, t1 was DSS-treated CreERT2 group, w1 was DSS-treated CreW group

In this study, rarefaction curve analysis for community richness showed that the sequencing volume covered all the microorganisms in the samples and met the data analysis requirements (Fig. 3). Based on these data, we found that the alpha diversity of the gut microbiota was markedly reduced in untreated CreERT2 mice compared with that in CreW mice, according to the Shannon and Simpson indices. DSS treatment disturbed and reduced the diversity of the intestinal microbiota. CreERT2 mice treated with DSS exhibited reduced ACE, Chao1, Observed-species, Shannon and Simpson indices compared with those of DSS-treated CreW mice (*P* < 0.05, Fig. 4A–E). This finding was also confirmed using rank-abundance curve (Fig. 5).

**Fig. 6:**
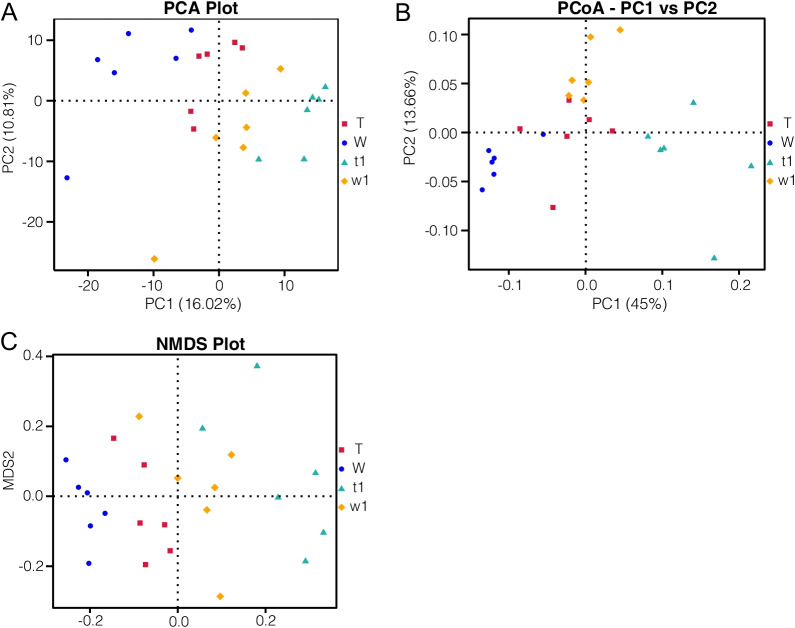
Comparison of beta diversity index between groups. Principal component analysis (PCA) indicated a symmetrical distribution of the gut microbial community among all the samples, which was also confirmed by principal coordinate analysis (PCoA) and nonmetric multidimensional scaling analysis (NMDS) (**A**–**C**). Note: n = 6; T was untreated CreERT2 group, W was untreated CreW group, t1 was DSS-treated CreERT2 group, w1 was DSS-treated CreW group

**Fig. 7:**
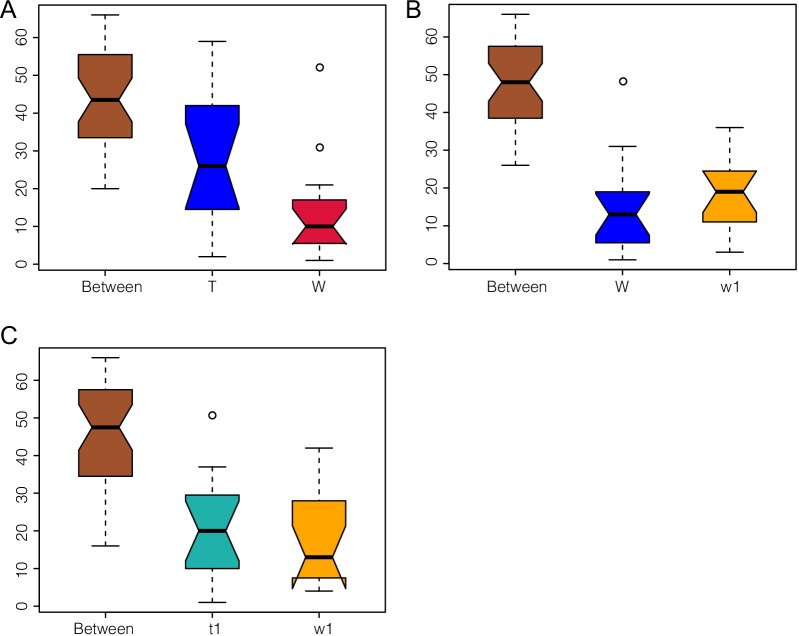
Anosim analysis indicated that the inter-group difference was significantly greater than the intra-group difference (**A**–**C**). Note: n = 6; T was untreated CreERT2 group, W was untreated CreW group, t1 was DSS-treated CreERT2 group, w1 was DSS-treated CreW group

Principal component analysis (PCA) showed that DSS caused changes in the baseline composition of the intestinal microbiota and revealed that the fecal bacterial community of DSS-treated or untreated CreERT2 mice was distinct from that of CreW control mice, which was also confirmed by principal coordinate analysis (PCoA) and nonmetric multidimensional scaling analysis (NMDS) (Fig. 6A–C). To further test whether there was a significant difference in the structural composition of the microbial community between DSS-treated and untreated CreERT2 and CreW mice, Anosim was conducted, showing that the differences between groups were greater than the differences within groups (Fig. 7A–C). The Adonis test derived from Bray–Curtis distance showed that the grouping factors contributed substantially to the OTU differences observed in this study (*P* < 0.05, Table 1). These results revealed that CreERT2 mice had a greater reduction in intestinal microbial diversity following DSS-induced colitis than CreW mice.

**Table 1:** Adonis analysis between groups

Compare	F value	R^2^	*P* value
W–T	5.7095	0.36344	0.001
W–w1	9.0254	0.47439	0.001
t1–w1	7.1184	0.41583	0.001

T was untreated CreERT2 group, W was untreated CreW group, t1 was DSS-treated CreERT2 group, w1 was DSS-treated CreW group

### Loss of Cldn7 alters the microbial composition of mice with colitis

**Fig. 8:**
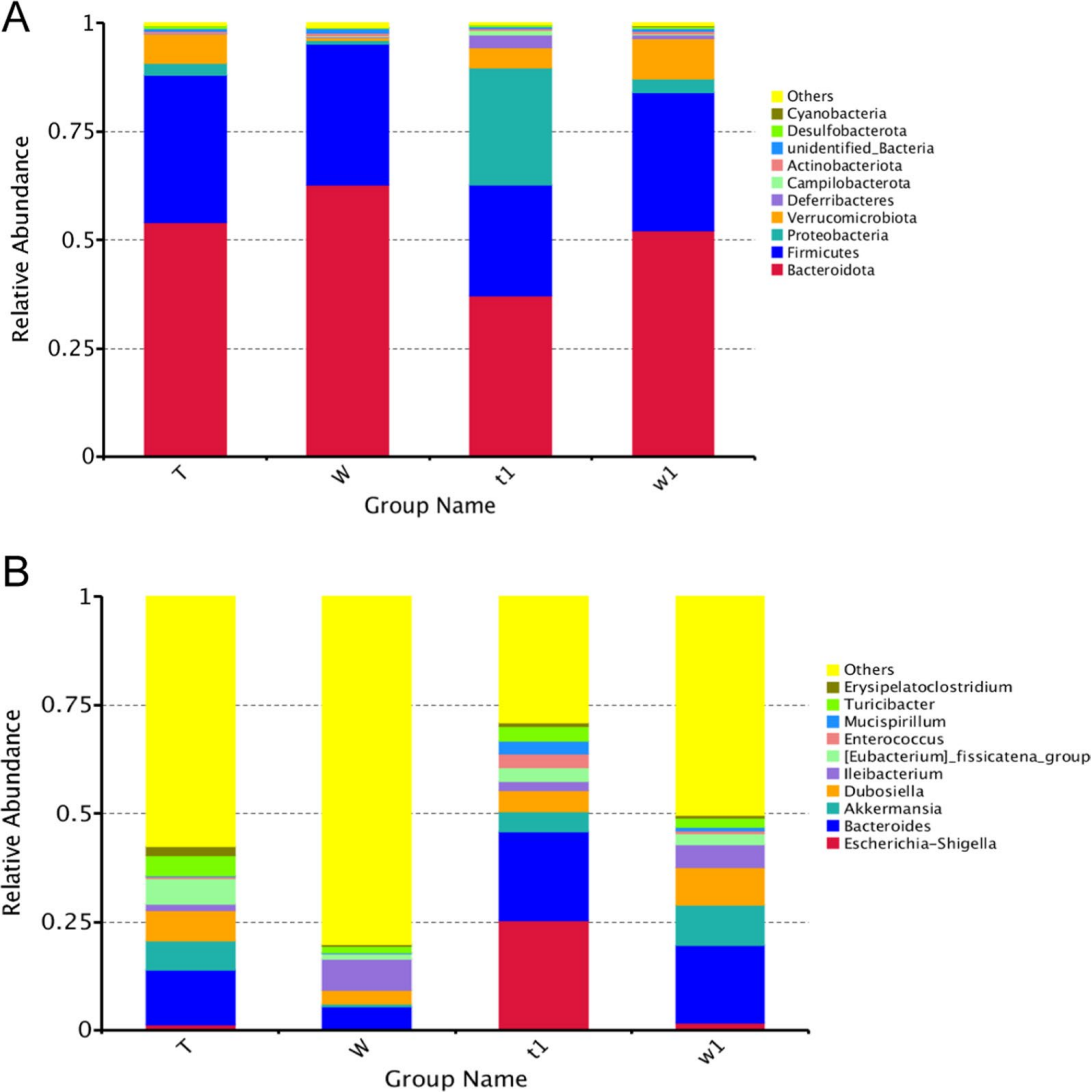
Relative abundance of intestinal microbiota at phylum level (**A**) and genus level (**B**) among groups. Note: n = 6; T was untreated CreERT2 group, W was untreated CreW group, t1 was DSS-treated CreERT2 group, w1 was DSS-treated CreW group

The community compositions of the intestinal microbes in untreated and DSS-treated CreERT2 and CreW groups were analyzed in different levels. The phyla *Bacteroidota* and *Firmicutes* were the most prevalent taxa in all groups, followed by *Proteobacteria*, *Verrucomicrobia* and *Deferribacteres*, while at the genus level, *Escherichia-Shigella*, *Bacteroides*, *Akkermansia*, *Dubosiella* and *Ileibacterium* were the top five genera (Fig. 8A, B).

**Fig. 9:**
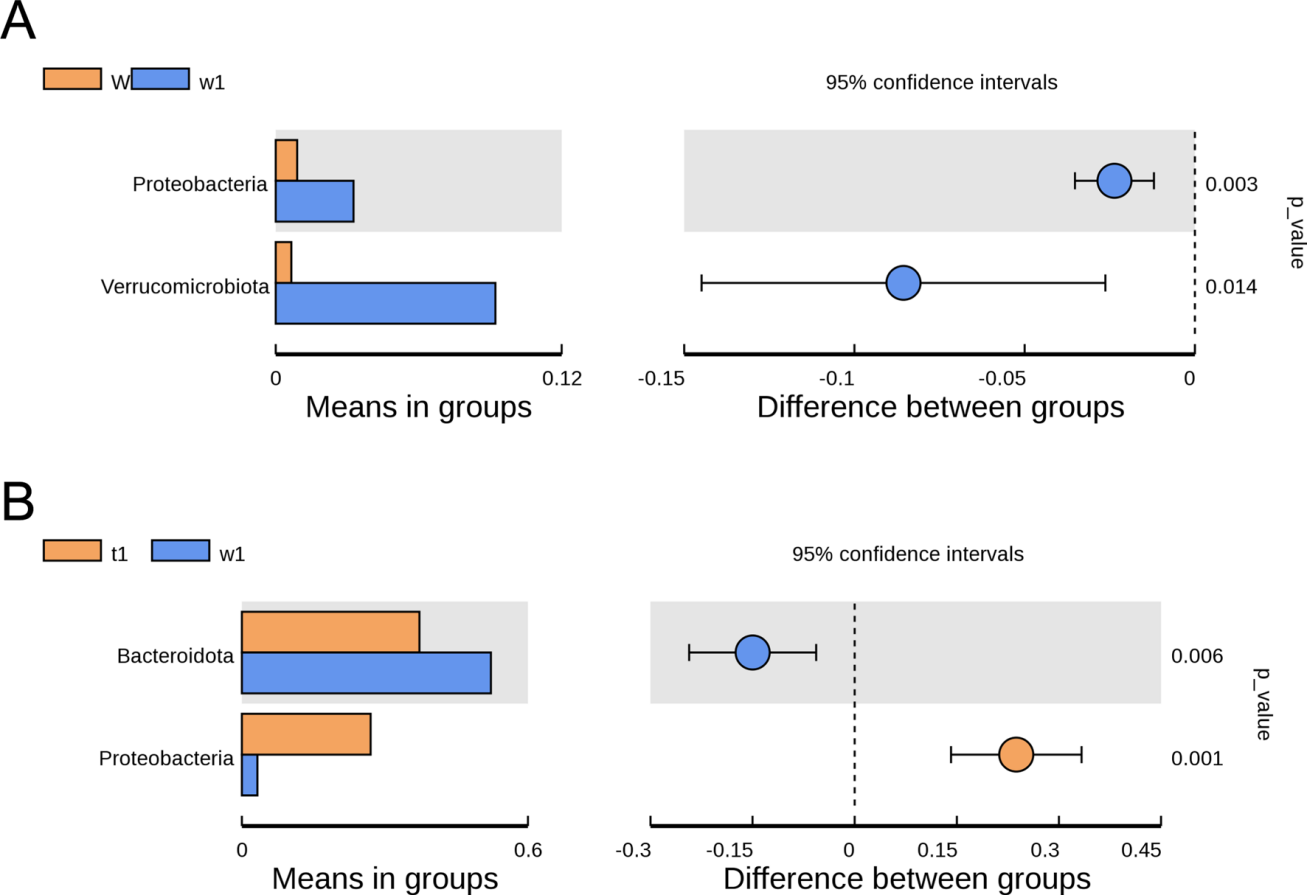
Differential bacteria divisions of intestinal microorganisms between two groups at phylum level (**A**, **B**). Note: n = 6; T was untreated CreERT2 group, W was untreated CreW group, t1 was DSS-treated CreERT2 group, w1 was DSS-treated CreW group

At the phylum level, the relative abundance of *Proteobacteria* and *Verrucomicrobia* in DSS-treated CreW control mice were significantly higher than those in CreW control mice (*P* < 0.05, Fig. 9A). After DSS treatment, the abundance of *Bacteroidota* in CreERT2 mice was markedly reduced, while these mice exhibited an increased abundance of *Proteobacteria* (*P* < 0.05, Fig. 9B).

**Fig. 10:**
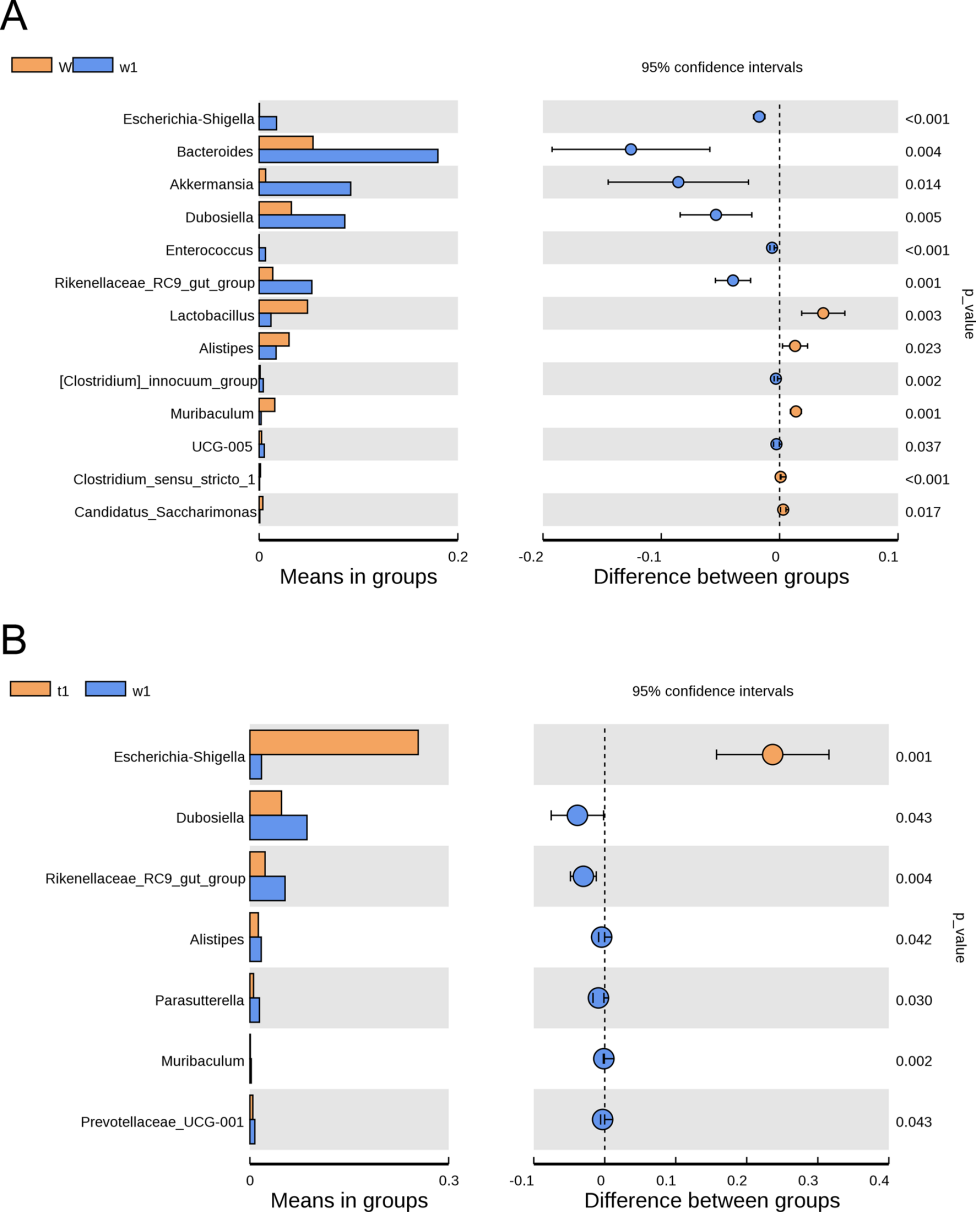
Differential bacteria divisions of intestinal microorganisms between two groups at genus level (**A**, **B**). Note: n = 6; T was untreated CreERT2 group, W was untreated CreW group, t1 was DSS-treated CreERT2 group, w1 was DSS-treated CreW group

At the genus level, the abundances of *Escherichia-Shigella*, *Bacteroides*, *Akkermansia*, *Dubosiella*, *Enterococcus*, *Rikenellaceae_RC9_gut_group*, *[Clostridium]_innocuum_group* and *UCG-005* were significantly higher in DSS-treated CreW mice than in untreated CreW mice (*P* < 0.05, Fig. 10A). DSS-treated CreERT2 mice also showed a higher abundance of *Escherichia-Shigella* than DSS-treated CreW mice (*P* < 0.05, Fig. 10B).

**Fig. 11:**
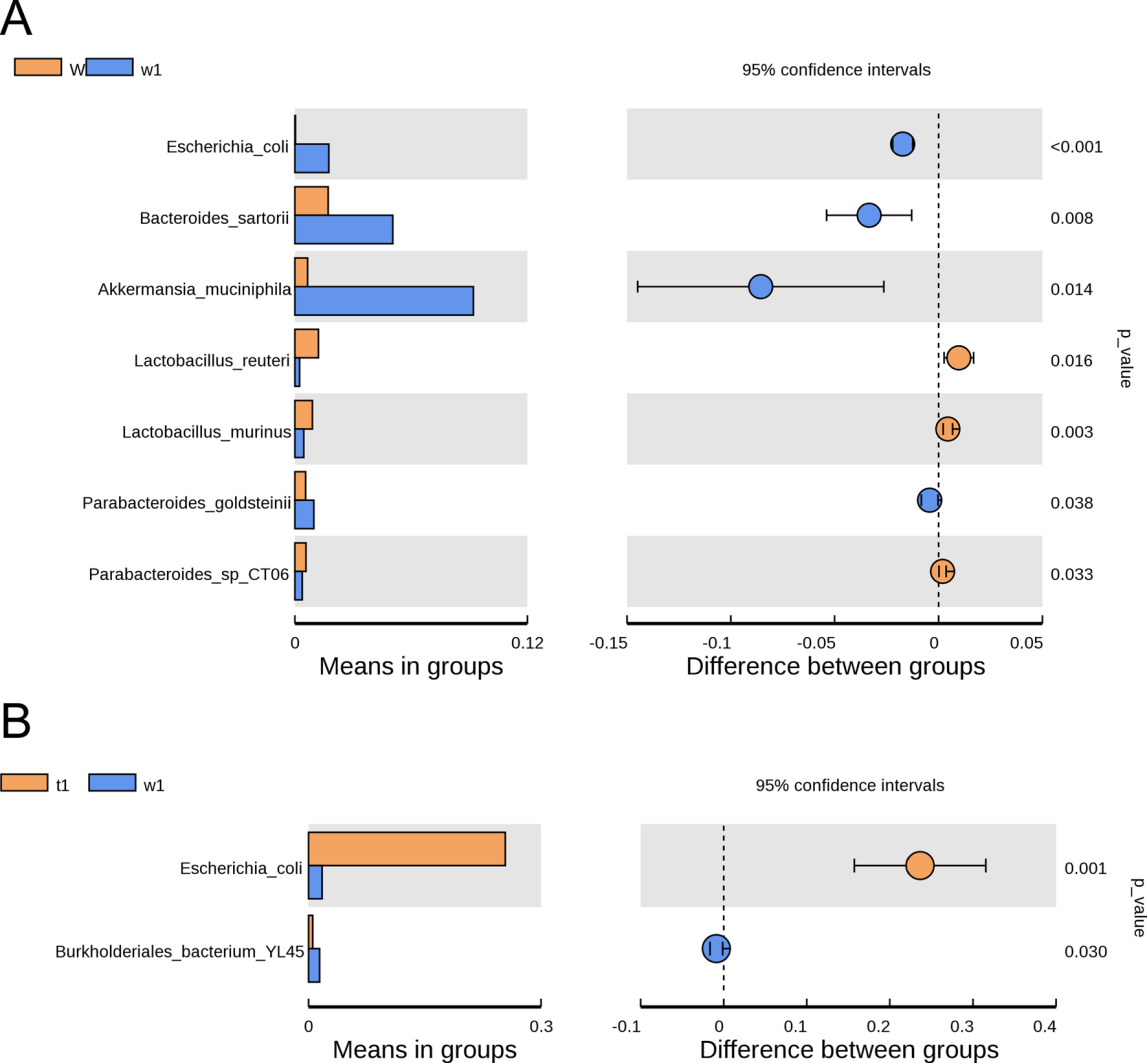
Differential bacteria divisions of intestinal microorganisms between two groups at species level (**A**, **B**). Note: n = 6; T was untreated CreERT2 group, W was untreated CreW group, t1 was DSS-treated CreERT2 group, w1 was DSS-treated CreW group

**Fig. 12:**
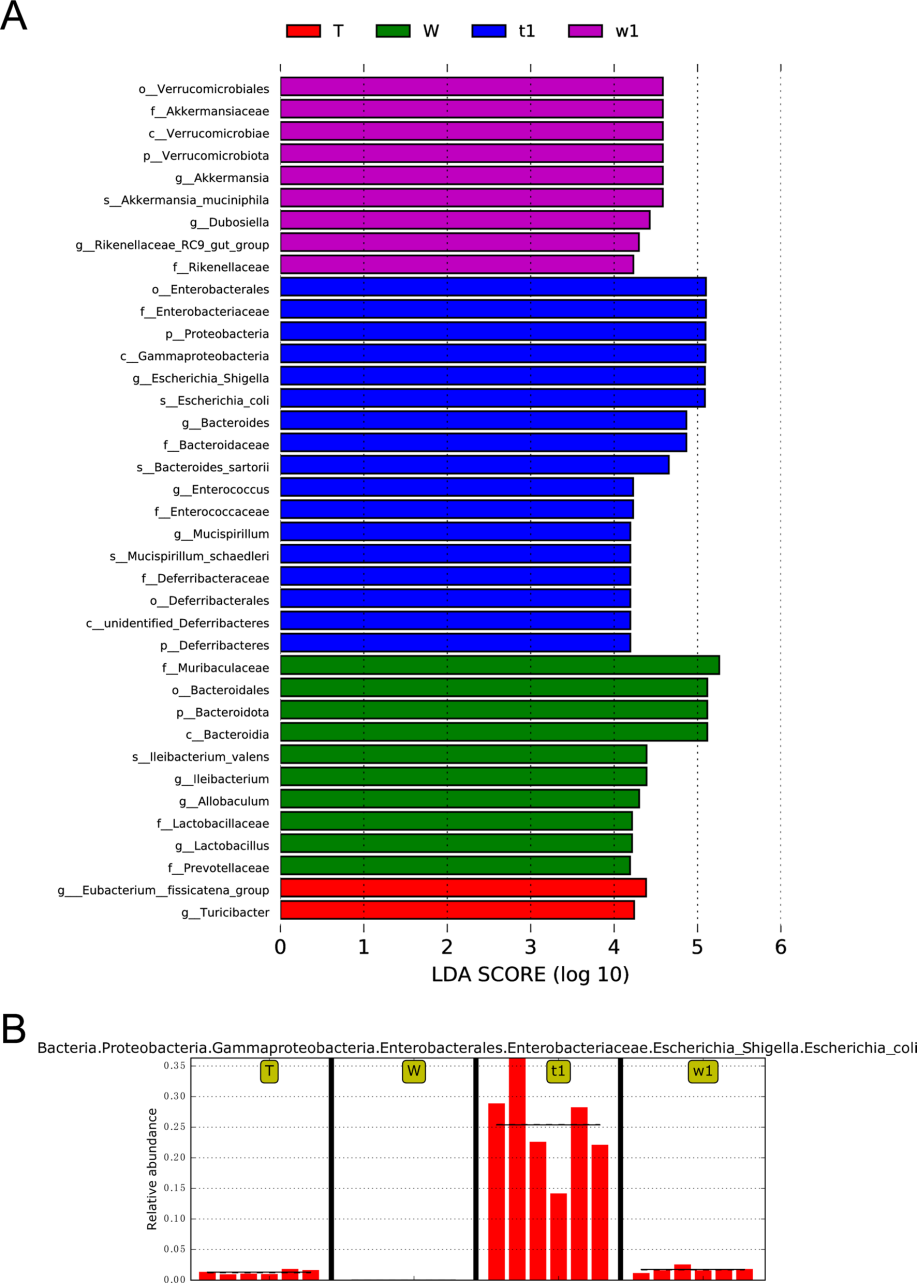
LEfSe analysis indicates the biomarkers of gut microbiota in untreated and DSS-treated CreERT2 and CreW mice. **A** The distribution histogram in LDA value showed the differentially abundant bacteria among groups. **B** Histogram of the relative abundance of *Escherichia coli* among groups. Note: n = 6; T was untreated CreERT2 group, W was untreated CreW group, t1 was DSS-treated CreERT2 group, w1 was DSS-treated CreW group

After observing on the phylum level and the genus level, we wonder whether there are differences at species level of bacterial composition between DSS-treated CreW and CreERT2 mice. As shown in Fig. 11A, *Escherichia_coli* (*E. coli*) was enriched after DSS exposure. Compared with that in CreW mice treated with DSS, CreERT2 mice showed a higher abundance of *E. coli* (Fig. 11B), indicating that Cldn7 knockout would cause a significant increase in the abundance of this species during DSS-induced colitis. To further explore these findings, LEfSe analysis was performed to obtain the biomarkers (the key bacterial members) of gut microbiota in untreated and DSS-treated CreERT2 and CreW mice. The results shown that *E. coli* (phylum *Proteobacteria*, genus *Escherichia_Shigella*) was the most significantly characteristic species in DSS-treated CreERT2 mice (Fig. 12A, B).

### Prediction of intestinal microbial function

**Fig. 13:**
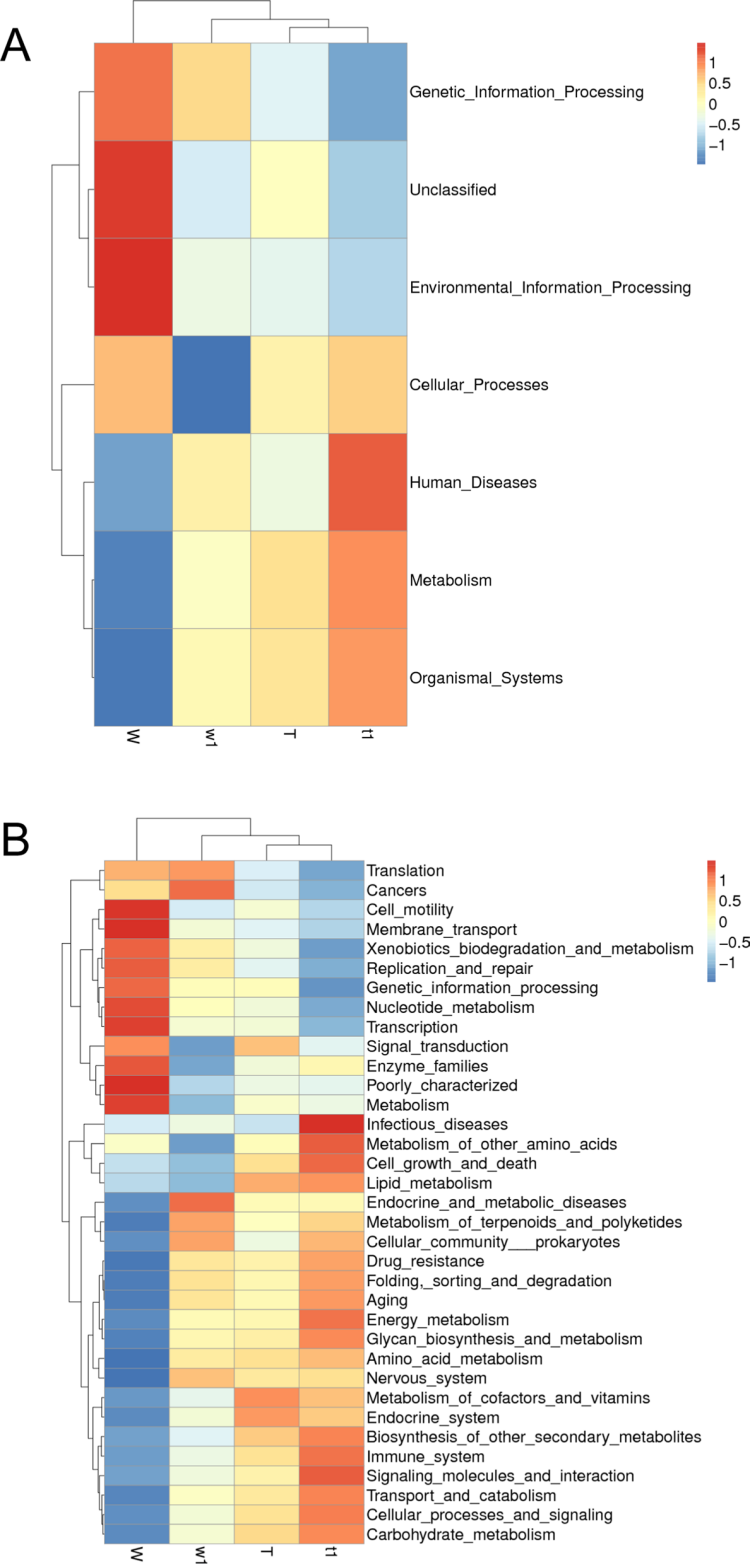
Prediction of intestinal microbial function. **A**, **B** The heatmap of predicted intestinal microbiota function in KEGG pathway at levels 1 (**A**) and 2 (**B**) in four groups [23–25]. Note: n = 6; T was untreated CreERT2 group, W was untreated CreW group, t1 was DSS-treated CreERT2 group, w1 was DSS-treated CreW group

Tax4Fun was applied to analyze the differences in KEGG pathways of the intestinal microbiota based on 16S rRNA gene amplicon sequencing profiling. Cluster analysis of the KEGG pathway [23–25] revealed that the gut microbes differentially expressed in the DSS-treated CreERT2 group are mostly representing the human diseases, metabolism and organismal systems pathways (level 1, Fig. 13A). Gene functions were classified into 35 KEGG pathways at level 2 revealing that there were obvious differences between DSS-treated CreW group and CreERT2 group in the heatmap (level 2, Fig. 13B). For human disease category, the pathway of infectious diseases was higher abundance in DSS-treated CreERT2 group. In metabolism category, the abundance in the pathways of other amino acids metabolism, lipid metabolism, energy metabolism and carbohydrate metabolism was higher in DSS-treated CreERT2 group than DSS-treated CreW group. In organismal systems, the pathway of immune system was higher abundance in DSS-treated CreERT2 group.

## Discussion

In our study, we successfully established experimental colitis in a mouse model using oral DSS administration. We demonstrated that the lack of IEC-specific Cldn7 aggravated the development of DSS-induced inflammation, with an increased DAI score, weight loss, colon length shortening, rectal bleeding and severe intestinal epithelial injury. Cldn7 deficiency has been reported to increase the paracellular flux of small organic solutes but not for the larger organic solutes, leading to increased infiltration of major bacterial products and aggravated intestinal inflammation [9]. Our previous studies have also shown that Cldn7 deficiency promotes intestinal inflammation by disrupting the integrity of TJs and increasing intestinal epithelial permeability. Epithelial hyperpermeability and microbial invasiveness have been long documented in IBD patients [26, 27]. Therefore, we investigated whether the intestinal barrier caused by the loss of Cldn7 would affect microbial invasion and thus promote the development of colitis.

The colonic mucus layer is continuously refilled by Muc2 mucin secreted by goblet cells, which is the first line of defence against bacteria, separating bacteria from epithelial cells and the host. Hence, mucus layer impairment allows commensal and pathogenic microorganisms to reach the intestinal epithelium, thereby leading to infection and inflammation [28]. We showed in this study that the lack of Cldn7 could disrupt the mucus layer by reducing the number of goblet cells and the secretion of Muc2 mucin, and promote translocation of intestinal bacteria, however, it does not regulate the total number of mucosal-associated bacteria during DSS-induced colitis. These findings indicated that the effect of Cldn7 on intestinal inflammation is not directly related to the total number of bacteria in the colonizing intestinal epithelium, but may be due to a shift in the balance between commensal and potentially pathogenic microorganisms [29].

Our analysis, using 16S rRNA sequencing, showed that CreERT2 mice had a greater reduction in colonic microbial diversity than CreW mice following DSS treatment. The diversity of microbial community contributes to the stability of the intestinal ecosystem and its ecological functions. The reduction in diversity could initiate an inflammatory response and promote the development of inflammatory diseases [30]. Our results showed that the loss of intestinal Cldn7 significantly reduced microbial diversity, suggesting that intestinal Cldn7 can maintain the stability of intestinal ecosystem. Therefore, Cldn7-deficient mice had lower stability of intestinal ecosystem, which would aggravate the development of DSS-induced inflammation.

The pattern of dysbiosis most associated with IBD patients is a decrease in commensal bacteria diversity, particularly in *Firmicutes* and *Bacteroidota*, and a relative increase of bacterial species belonging to *Proteobacteria* [31]. Consistent with the results, we also found a significant increase in *Proteobacteria* and a slight decrease in *Firmicutes* and *Bacteroidota* in CreW mice after DSS exposure, which was further enhanced in CreERT2 mice. Here, we showed that DSS-treated CreERT2 mice had higher relative abundance of *E. coli*. Furthermore, LEfSe analysis indicated that *E. coli* may be the key bacteria in Cldn7 knockout mice during DSS-induced colitis.

*E. coli*, belonging to the phyla *Proteobacteria*, has been highlighted in initiating and maintaining the inflammatory process in IBD gut tissues [32]. *E. coli* is a gram-negative aerobic bacterium, and most intracellular *E. coli* have no characteristic pathogenic characteristics. However, *E. coli* with an adherent and invasive pathotype (AIEC) has the ability to adhere to and invade IECs and to replicate within macrophages and induce TNF-α secretion without inducing cell apoptosis, which damages the intestinal mucosal barrier and further triggers the massive release of proinflammatory factors [33]. Abnormal mucosal immunity or intestinal barrier dysfunction is an important factor for AIEC to invade mucosal cells and promote inflammation [34]. It is reported that approximately 40% of CD patients have overrepresented AIEC compared to healthy controls [35]. The prevalence of AIEC in UC is not as clear as that in CD. Some studies have found an increased abundance of *E. coli* in active UC lesions compared with inactive mucosa or healthy people [36]. Although it is not clear whether AIEC causes intestinal inflammation, which can lead to disease; or whether AIEC overgrowth in the intestinal mucosa could be a consequence of preceding dysbiosis and/or intestinal inflammation and lead to a worsening of disease [37]. In our constructed intestinal-specific Cldn7 knockout mice, tamoxifen-induced intestinal Cldn7 deletion led to intestinal inflammation, mucus layer impairment and microbiota dysbiosis. Therefore, we speculate that pre-existing dysbiosis and gut inflammation in intestinal Cldn7 knockout mice could promote the invasion and proliferation of *E. coli* on the intestinal mucosa, further damage the intestinal barrier, induce microbiota dysbiosis and aggravate the development of DSS-induced colitis. Nevertheless, it is not clear exactly how *E. coli* causes intestinal inflammation, but it can be assumed that the destruction of the intestinal epithelial barrier leads to the imbalance of the mucosal immune system and the intestinal microbiota.

This disruption in the gut microbiota balance ultimately resulted in an alteration of the gut micro flora-associated functions, including alteration of human disease such as infectious diseases pathway, alteration of metabolism such as energy, lipid and carbohydrate pathways, and organismal systems alterations, such as immune system imbalance. Upregulated pathway of infectious diseases in DSS-treated CreERT2 group may be related to the clinical pathogenicity of *E. coli.* The high nutrient competition between the commensal microbiota and *E. coli* pathobiont requires the latter to occupy its own metabolic niches to survive and proliferate in the intestine [38], so the increased energy, lipid and carbohydrate metabolism pathways could provide energy benefits for *E. coli* and promote its colonization. In the mouse model of experimental colitis, the lack of intestinal Cldn7 caused intestinal epithelial dysfunction and dysbiosis. This disruption of intestinal homeostasis would lead to the activation of the immune system [39].

Fecal samples are often used as substitutes for the gut microbial contents because they are relatively easy to collect in clinical laboratories [15]. Nevertheless, the composition of mucosa-associated bacteria was reported to differ from that of fecal microbiota, and may better reflect regional changes in gut microbes at mucosal surfaces at sites of inflammation [40, 41]. Further studies, exploring mucosa-associated microbial population in a larger study will yield further information.

## Conclusions

Collectively, our results for the first time suggested an association between intestinal epithelial Cldn7 knockout and microbiota dysbiosis from the perspective of microbial population, diversity, spatial, number and function changes during inflammatory events. This study may contribute to understanding the interaction between gut microbiota, epithelial cells and colitis, which will provide a basis for the study of the epithelial barrier impairment affecting intestinal microorganisms to promote inflammation.

## Data Availability

The datasets used and analysed during the current study are available from the corresponding author on reasonable request.

## Abbreviations

IBD: Inflammatory bowel disease
IECs: Intestinal epithelial cells
TJs: Tight junctions
Cldn7: Claudin-7
DSS: Dextran sulfate sodium
DAI: Disease activity index
FISH: Fluorescence in situ hybridization
OTUs: Operational taxonomic units
LEfSe: Linear discriminant analysis Effect Size
KEGG: Kyoto Encyclopedia of Genes and Genomes
CreERT2 mice: Inducible intestinal conditional Cldn7 gene knockout mice
PCA: Principal component analysis
PCoA: Principal coordinate analysis
NMDS: Nonmetric multidimensional scaling analysis
Anosim: Analysis of similarities
*E. coli*: *Escherichia coli*
AIEC: Adherent and invasive *Escherichia coli*
